# Quercetin in COPD: A Multi-Target Approach to Modulate Inflammation, Oxidative Stress, and Epithelial Dysfunction

**DOI:** 10.3390/ijms27156548

**Published:** 2026-07-23

**Authors:** Priyanka Sarkar, Umadevi Sajjan

**Affiliations:** 1 Center for Inflammation and Lung Research, Lewis-Katz Medical School, Temple University, Philadelphia, PA 19140, USA; priyanka.sarkar@temple.edu; 2 Department of Microbiology, Immunology and Inflammation, Lewis-Katz Medical School, Temple University, Philadelphia, PA 19140, USA; 3 Department of Thoracic Medicine and Surgery, Temple University Health System, Philadelphia, PA 19140, USA

**Keywords:** COPD, Quercetin, anti-inflammation, antioxidant, anti-viral

## Abstract

Chronic obstructive pulmonary disease (COPD) is a progressive lung disorder that affects millions of people globally. Although the mechanisms of COPD pathogenesis are not completely known, oxidative stress and lung inflammation caused by chronic exposure to cigarette smoke, environmental or occupational pollutants, gas from burning biomass fuel are thought to contribute to development of COPD. Therefore, therapies aimed at reducing oxidative stress along with inflammation may be important in treating COPD. However, the current pharmacological therapies treat symptoms and reduce acute exacerbations, but do not treat the root cause of COPD. Quercetin is a plant polyphenol present in berries, apples and onions, and has potent antioxidant and anti-inflammatory properties. Quercetin inhibits oxidative stress by scavenging reactive oxidant species and promoting expression of antioxidant enzymes. It reduces inflammation by inhibiting various kinases that participate in the expression of pro-inflammatory cytokines. It also alters gene expression by functioning as an epigenetic modifier. Quercetin also acts as antiviral agent by attenuating viral entry and replication. In preclinical models of COPD, quercetin reduces oxidative stress, lung inflammation, goblet cell metaplasia, expression of matrix metalloprotease MMP-9 and MMP-12, and prevents rhinovirus-induced progression of emphysema. It also promotes normal regeneration of airway epithelium by improving cell polarization, reducing goblet cell hyperplasia and increasing number of ciliated cells. This review compiles the current understanding of the biological properties of quercetin and its potential therapeutic role in COPD. We also summarize its potential benefits over the current therapeutic drugs used to treat COPD.

## 1. Introduction

Quercetin is a naturally occurring flavonol, a subgroup of flavonoids. It is found in fruits, vegetables, and grains such as apples, red onions, berries, and leafy greens like lettuce [1,2]. Quercetin has gained considerable attention due to its potent antioxidant and anti-inflammatory properties. Its antioxidant property is due to its ability to scavenge reactive oxygen species (ROS), regulate cellular redox balance by modulating glutathione levels and increasing the expression of antioxidant enzymes [3,4,5,6,7]. Its anti-inflammatory effect mostly arises from inhibiting protein and lipid kinases required for activation of several transcription factors that promote expression of pro-inflammatory cytokines [8,9,10,11,12,13,14,15,16,17]. Quercetin has also been shown to exert antimicrobial activities against bacteria, fungi, and viruses [18,19,20,21,22,23,24,25]. Apart from these well-studied properties, recent findings also indicate quercetin inhibits methyltransferases thus capable of modulating epigenetic changes [26,27,28]. Through these mechanisms, quercetin has been demonstrated to have protective effects in multiple diseases associated with oxidative stress, chronic inflammation, infection and gene dysregulation due to epigenetic modulation [29,30,31,32,33,34].

Chronic obstructive pulmonary disease (COPD) is a progressive respiratory disorder characterized by persistent airflow limitation, chronic airway inflammation, and structural remodeling of lung tissue. It is the third worldwide cause of death [35]. COPD is primarily caused by long-term exposure to cigarette smoke, environmental pollutants, and other inhaled irritants that trigger persistent oxidative stress and inflammatory responses within the airways and lung parenchyma [36]. A hallmark of COPD pathogenesis is excessive oxidative stress, resulting from the overproduction of reactive oxygen species and an imbalance between oxidants and antioxidants [37]. These processes initiate the characteristic inflammatory signature of COPD by activating inflammatory cells such as neutrophils and macrophages, leading to the release of cytokines, chemokines, and proteolytic enzymes that contribute to airway remodeling, mucus hypersecretion, and destruction of alveolar structures [38,39]. Unlike in asthma, the inflammation is corticosteroid resistant [40,41]. However, a corticosteroid responsive eosinophilic inflammation exists in a subset of COPD patients referred to as having type-2 (T2) inflammation. Some studies have shown that approximately 10–40% of COPD patients exhibit features of eosinophilic or T2-high inflammation, which is associated with increased exacerbation risk and distinct therapeutic responses [39].

Acute exacerbations are the major cause of morbidity and mortality in COPD patients and are usually diagnosed by increased respiratory symptoms, such as dyspnea, a more severe cough and increased sputum production [42,43,44,45]. On average, COPD patients experience 1 to 4 acute exacerbations per year [46,47]. Majority of acute exacerbations are associated with respiratory infections caused by either bacteria, virus or both [48,49,50]. Approximately 20 to 30% of acute exacerbations are bacteria-associated with more common being non-typeable *H. influenzae*; 20 to 50% of the acute exacerbations are related to viral infections with rhinovirus being more common; approximately 25% are associated with both bacterial and viral infections [48,49,50,51]. In some patients, bacteria colonize and persist as biofilm [52,53,54].

Oxidative stress, chronic lung inflammation and respiratory infections play central roles in COPD pathogenesis. Therefore, naturally occurring compounds like flavonoids with potent antioxidant, anti-inflammatory and antimicrobial properties have attracted increasing interest as potential therapeutic agents [55,56,57]. Among these, quercetin has emerged as a promising candidate due to its ability to modulate multiple molecular pathways involved in lung inflammation and oxidative injury. Its anti-inflammatory and antioxidant properties help to reduce the risk of chronic respiratory diseases like pulmonary fibrosis and COPD [58].

Given its ability to modulate multiple pathogenic mechanisms including oxidative stress, inflammatory and immune responses, and airway remodeling, quercetin may represent a promising therapeutic candidate for COPD. In this review we summarize the current understanding of the biological properties of quercetin and its potential therapeutic role in COPD.

## 2. Methodology

This review article was compiled using a narrative review approach to summarize the current understanding of the therapeutic potential of Quercetin in COPD treatment in comparison to the available treatment options of COPD. Relevant articles were identified through PubMed and google Scholar searches up to June 2026. The following key words were used during the search process: anti-inflammatory effect of quercetin in epithelial cells/bronchial epithelial cells, antioxidant effect of quercetin in epithelial cells/bronchial epithelial cells, quercetin role in COPD, viral exacerbation in COPD, anti-viral effect of quercetin, treatment of COPD.

Original research articles, review articles, preclinical studies, and clinical studies relevant to the role of quercetin in COPD pathogenesis, oxidative stress, inflammation, epithelial dysfunction, and respiratory viral infections were included. Studies not available in English, unrelated to respiratory disease and/or epithelial cells, or lacking sufficient relevance to quercetin-mediated mechanisms were excluded.

The selected literature was critically evaluated and organized into sections focusing on COPD pathophysiology and available treatments, antioxidant and anti-inflammatory properties, and antiviral activity of quercetin, bioavailability and formulation challenges of quercetin, and current evidence supporting its therapeutic potential in COPD.

## 3. Quercetin Chemistry and General Properties

### 3.1. Quercetin Chemistry

**Figure 1:**
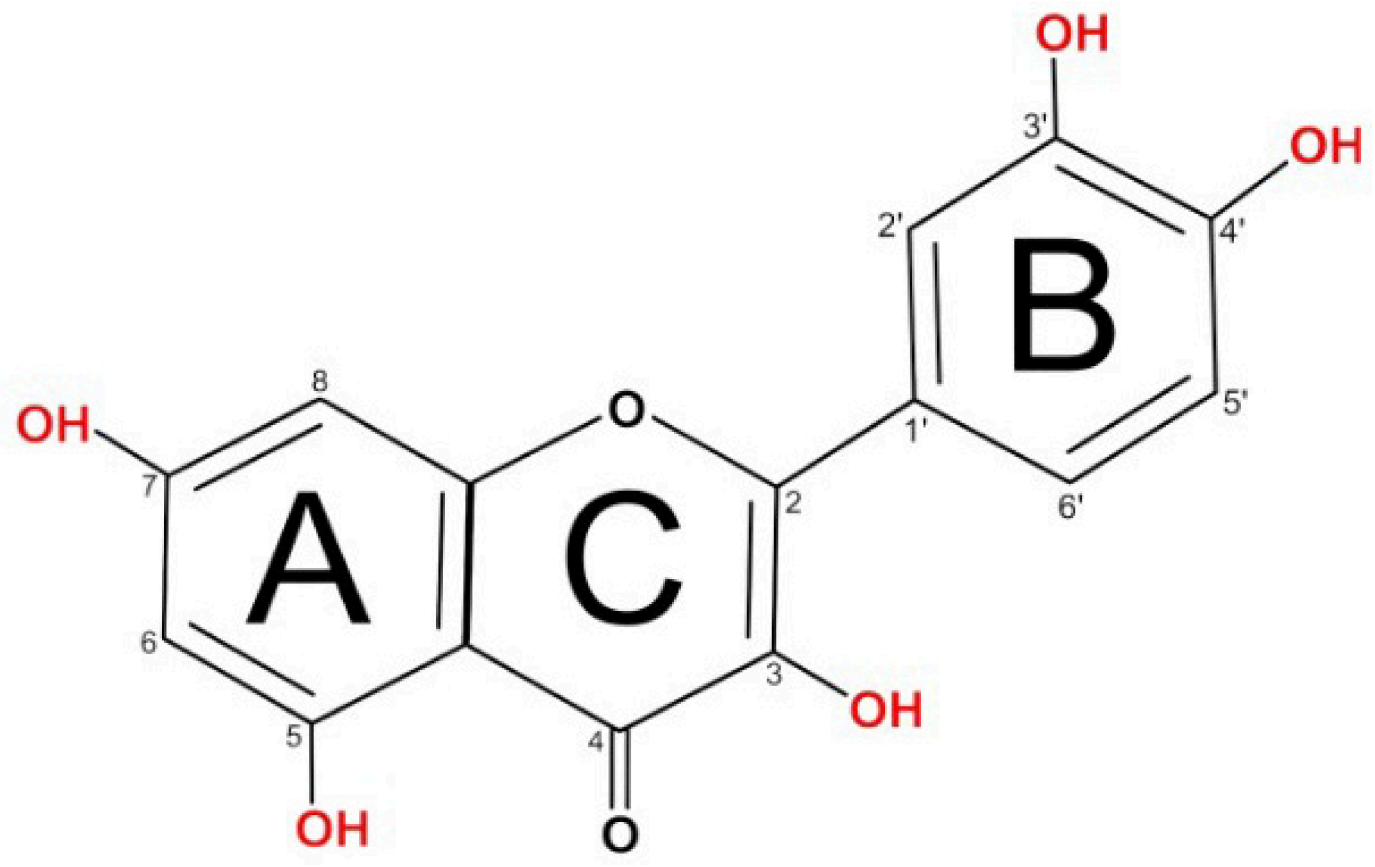
Adapted from Carrillo-Martinez [61] and drawn in Chemdraw. The figure shows the chemical structure of quercetin with two phenolic rings (A and C) and one catechol ring (B) with most important being catechol ring with 2 hydroxyl groups in the neighboring positions. The phenolic hydroxyl groups of quercetin (marked in red color) act as donors of electrons, and these are responsible for the capture activity of free radicals. Catechol ring supported by the double bond (between position 2 and 3)-gives quercetin its antioxidant capacity.

Quercetin is a major polyphenolic flavonoid found in multiple fruits and vegetables. Figure 1 shows the chemical structure of quercetin. Quercetin is characterized by 15 carbon atoms arranged in a C6-C3-C6 configuration with 2 phenolic rings (A and B rings) connected to a heterocyclic pyranone/catechol ring (C ring) creating a planar conjugated structure. Additionally, quercetin also has a double bond between position 2 and 3 carbon of the C ring, a feature of flavonols. The hydroxyl groups at position 3 and 5, and 2,3-double bond conjugated with a 4-oxo group at position 4 contributes to stability and antioxidant properties of quercetin. Furthermore, quercetin has 5 hydroxyl groups (Figure 1, highlighted in red), which determines the possible number of derivatives. These hydroxyl groups along with the catechol structure (3′,4′-dihydroxy group) on the B ring enhances the radical scavenging ability, metal ion chelating ability and redox activity. This pattern of substitution enable quercetin to participate in hydrogen bonding and electron transfer reactions, mechanisms underlying its anti-inflammatory activity [59,60,61].

Plants synthesize quercetin in the form of aglycone, quercetin glycoside and methylated quercetin [60,62]. The most common naturally occurring form is 3-O-glucoside also known as isoquercetin, rutin which is quercetin linked with two sugar residues rhamnose at C-3 and glucose at C-6 position, and hyperoside, quercetin-3-O-galactoside [63,64,65]. Some medicinal plants also synthesize methylated form of quercetin (4′-O-methylquercetin) termed as tamarixetin [66,67].

### 3.2. Bioavailability and Pharmacokinetics of Quercetin

Bioavailability is the fraction of the ingested compound that is absorbed and distributed to be available at the place of action. The bioavailability from animal studies cannot be extrapolated to humans, because of the quercetin dose used in animals and differences in enzymes and digestive processes. Therefore, this section will focus on bioavailability of quercetin in humans. The bioavailability of quercetin aglycone is relatively low that is approximately 2% after a single oral dose 4 g in a human study [68] and this is attributed to its hydrophobicity, stability and absorption profile. It is thought that due to its hydrophobic nature, enterocytes in the small intestine absorb quercetin aglycone by simple diffusion and by pH-dependent organic anion transporter B [2,69]. In contrast, the glycoside derivatives of quercetin, such as rutin and isoquercetin are absorbed by the gut better than aglycone form due to their solubility in water [70]. The quercetin glycosides are transported into the enterocytes through sodium-dependent glucose transporter (SGLT)1. But major portion of the orally administered quercetin glycoside is converted to aglycone by enzymatic action of lactase phlorizin hydrolase and cytosolic β-glucosidases in the small intestine or microbial β-glucosidases and α-rhamnosidase in the colon, and absorbed by enterocytes as quercetin aglycone [71]. Compared to quercetin aglycone, glycosylated quercetin is rapidly absorbed, that is within 30 min after oral ingestion in healthy human subjects [72,73]. However, the maximum plasma levels observed were 2 to 5 µmol/L based on the glycosides tested.

Therefore, investigators have developed various formulations of quercetin to enhance the bioavailability. Addition of oligosaccharide (9 glucose residues) enhances the bioavailability by 2 fold compared to isoquercetin in healthy subjects [74]. Guo et al. found that patients with low levels of plasma Vitamin C are inefficient in absorbing quercetin. Oral administration of quercetin along with equal amount of Vitamin C increased bioavailability and reduced the low density lipoprotein levels [75]. Oral treatment with quercetin aglycone (500 or 1000 mg/day) with Vitamin C and niacin for 12 weeks increased the quercetin levels in plasma to high µM levels (400 to 600 µM) [76].

Bioavailability increases when quercetin is administered orally in plant matrix or when emulsified with lipid molecules into nanoparticles or phytosome. For example, oral administration of onion skin extract powder or apple peel extract containing quercetin was absorbed 1.4 and 5 times better than quercetin dihydrate [77,78]. Quercetin emulsified with galactomannans, lecithin and sunflower oil or mixed with γ-cyclodextrins and administered in capsules increased the bioavailability by 21.7 to 29.2 folds compared with quercetin dihydrate [79,80]. In another study, oral administration of lecithin based quercetin dihydrate containing phytosome was shown to increase plasma quercetin levels 100 to 150 fold compared to quercetin dihydrate [81]. Based on these observations, there are several reports developing quercetin containing lipid nanoparticles to improve the bioavailability of quercetin. However, the toxicity of the long-term administration of quercetin nanoparticles or phytosome is yet to be determined in human subjects.

After absorption, quercetin undergoes phase II metabolism in both small intestine and liver to generate sulphated, methylated or glucuronide conjugates. Metabolism of quercetin is catalyzed by the action of sulfotransferases, uridine-5′-diphosphate glucuronosyl transferases (UGTs), and catechol-*O*-methyl- transferases (COMTs) [82]. Metabolites transported from the intestine and those generated in liver enter the blood stream. Less than half of the metabolites are also secreted into the bile, which is then excreted in the feces. The primary circulating metabolites in the bloodstream of humans are quercetin-3-glucuronide, quercetin-3′sulfate and methyl derivatives such as isorhamnetin [83,84].

Pharmacokinetic profile of quercetin depends on the route of administration, whether quercetin is administrated in the form of aglycone, as derivatives, in their natural plant matrix or in formulation, and the dose of quercetin. Pharmacokinetics profiles compiled from the published studies are presented in Table 1. Pharmacokinetic profiles also varied from one study to another even though similar levels of quercetin was used. For example, quercetin aglycone at 500 mg/day showed variable T_max_ (3–4.83 h), T_1/2_ (3.5–8.3 h) and C_max_ (10.9 to 1051 ng/mL). The variability in C_max_ appear to be due to co-administration of quercetin aglycone with other substances rather than fed [73,85] or fasting state [86], or single or multiple doses. Higher C_max_ was observed when quercetin was mixed with tang or incorporated into First strike bars, both of which contains mixture of vitamins, sugars, guar and xanthan gums in addition to other excipients, and RealFX-Q plus chews which has Vitamin C and lipids/fats [86], compared with quercetin administered alone [85]. While the lipids and fats emulsify the quercetin, guar and xantham gums maintain the dispersion of quercetin, and this may facilitate the absorption. The highest C_max_ observed with consuming quercetin chews and quercetin in bars was also attributed to absorption by both buccal and gastrointestinal route, because these formulations require chewing. In addition, quercetin aglycone is absorbed by passive diffusion and pH-dependent organic anion transporter B. Polymorphisms in organic anion transporter B can influence absorption of quercetin indicating pharmacokinetics of quercetin may also depend on the subjects recruited in the study [69,87]. Pharmacokinetic profile of quercetin administered as glycosides also varies from one study to another and seem to depend on the type of sugar substitutions. Quercetin administered as phytosome, had better pharmacokinetic profile compared to quercetin, but the C_max_ was lower than quercetin administered in its own matrix such as onion and apple extracts [81] However the total quercetin absorption per hour was much higher with phytosome formulation. Intriguingly quercetin glucosides showed a lower t_1/2_ and higher C_max_ than quercetin aglycone and this may be due to hydrophilic nature of glucosides facilitating its dispersion and absorption in the small intestine directly by SGLT-1 or after catalysis by enzymes as aglycone. Quercetin administered in its natural plant matrix, for example onion extracts showed similar T_max_ despite dose of quercetin in the onion extracts, but the T_1/2_ and C_max_ varied with the dose [73,88]. Pharmacokinetic profile and bioavailability may also depend on the gastrointestinal health of subjects since oral administered quercetin given in any form depends on processing and absorption by the gut. Overall, quercetin administered as glucosides or in plant matrix appears to have better pharmacokinetics and absorbed more efficiently than quercetin aglycone.

### 3.3. Antioxidative Role of Quercetin

The biological activity of quercetin is largely driven by its antioxidant properties. It is a potent antioxidant flavonoid, highly valued as a therapeutic molecule against free radicals. Its antioxidant activity is closely linked to its chemical structure, particularly the catechol moiety, the hydroxyl substitutions, and the conjugated double bond system, which together facilitate free radical scavenging, electron donation, and metal chelation (Figure 1) [90]. The antioxidant role of quercetin against free radicals such as 1,1-diphenyl-2-trinitrophenylhydrazine (DPPH), hydroxyl radicals, nitric oxide, hydrogen peroxide radicals, and superoxide was demonstrated by reducing power assay [91]. In another study, free radical scavenging by quercetin and quercetin-DNA complex was compared using DPPH and thiobarbituric acid–reactive substances (TBARS) [92]. Quercetin-DNA complex was found to be much stronger than quercetin in scavenging the DPPH and TBARS. Moreover, quercetin also protected DNA from free radical damage and that the DNA-quercetin complex has 80% of DPPH inhibition capacity. Quercetin has also been shown to protect neuronal cells from reactive oxygen species generated by hydrogen peroxide and prevent apoptosis [93].

Beyond its direct neutralizing effect on reactive oxygen species and reactive nitrogen species, quercetin also modulates intracellular antioxidant defense systems by activating redox-sensitive cytoprotective pathways like activating the Nrf2 signaling axis [94]. Activation of Nrf2 by quercetin promotes the transcription of antioxidant response element (ARE)-dependent genes, like hemoxygenase (HO)-1, (NAD(P)H quinone dehydrogenase 1 (NQO1), Glutamate-Cysteine Ligase (GCL), superoxide dismutase (SOD), catalase, and glutathione-related enzymes (GPX), thereby enhancing endogenous cellular defense against oxidative injury. Quercetin also induce antioxidant enzymes, such as catalase, peroxiredoxins, glutathione peroxidases by transcriptionally activating FOX3A in liver cells [95]. Quercetin not only enhances the production of HO-1 and catalase but also interacts with them to enhance their free radical scavenging activity. Similarly, quercetin interacts with glutathione peroxidases to enhance its antioxidant capacity [94,96].

This antioxidant activity is functionally important because oxidative stress not only damages cellular macromolecules but also amplifies inflammatory signaling, mitochondrial dysfunction, and tissue injury in diverse pathological settings.

### 3.4. Anti-Inflammatory Role of Quercetin

Inflammation is required for innate and adaptive innate immune response to clear the irritant stimuli or pathogens. But exaggerated and persistent inflammation leading to tissue degradation is detrimental and may lead to the development of chronic diseases. In addition, the persistent oxidative stress stimulated by exogenous or endogenous stimulation also leads to inflammation. Lipid and protein kinases play a pivotal role in activating almost all the inflammatory signaling pathways. Quercetin exerts anti-inflammatory activity not only by inhibiting oxidative stress but also through suppression of major pro-inflammatory signaling pathways. Its anti-inflammatory effect has been studied in many diseases and is known to suppress the expression and release of multiple key players of inflammation [9]. The anti-inflammatory property of quercetin is thought to be due to its ability to compete for adenosine tirphophate (ATP) binding site in various protein and lipid kinases, thus inhibiting the activity of these enzymes [97]. In line with this notion, quercetin prevents lipopolysaccharide (LPS)-induced inflammation by inhibiting Src and Syk-mediated PI3-kinase activation in macrophages [98]. LPS can also induce cyclooxygenase-2 (COX-2), inducible nitric oxide synthase (iNOS), and cytokines via activation of MAP kinase and P300 signaling, and this is reduced by quercetin [71,99]. Quercetin also inhibits TLR4 activation, thus preventing downstream activation of NF-κB and expression of inflammatory mediators and cytokines in dendritic cells [100]. Quercetin inhibits Caspase-1 expression and the activation of NLRP3 and AIM2 inflammasomes thus reducing the secretion of mature IL-1β [101]. It also suppresses inflammation-induced Iκκ-α/β and iNOS in the liver of diabetic rats [102]. Quercetin has also been reported to influence innate immune-cell polarization and inflammatory cell behavior, for example it attenuates macrophage polarization towards inflammatory M1 phenotype thus promoting an anti-inflammatory transition [103]. These findings support quercetin as a multi-target bioactive molecule capable of regulating oxidative stress and inflammation simultaneously, although its biological efficacy in vivo remains strongly influenced by formulation, metabolism, and bioavailability.

### 3.5. Quercetin as an Antiviral Agent

Quercetin has been shown to act as antiviral agent against both DNA and RNA viruses and also non-enveloped RNA viruses. In this review we will focus on respiratory viruses. Several forms of quercetin have been reported to act against multiple respiratory viruses like influenza, metapneumovirus, coronavirus, rhinovirus, and respiratory syncytial virus (RSV) [104] (Table 2). Quercetin and its derivatives appear to exert its antiviral effect mainly by inhibiting the viral entry into the cells followed by viral replication within the cells, thus inhibiting the host-driven inflammatory responses. Isoquercetin was found to be more effective than quercetin in inhibiting influenza virus infection [105]. Influenza viruses bind to and enter the cells by the action of neuraminidase and matrix 2. Molecular docking studies indicate quercetin bind neuraminidase and matrix 2 comparable to or more avidly than existing anti-influenza drugs, oseltamivir and zanamivir which target neuraminidase and matrix 2 respectively [106,107]. Quercetin and quercetin glucosides also reduce viral replication by inhibiting viral polymerase [108]. In mouse models of influenza virus infection, treatment with quercetin or quercetin derivatives decreased the mortality, reduced the levels of IFN-γ, iNOS, and RANTES in the lungs and delayed the development of progression of pulmonary lesions [109,110].

Quercetin can also block coronavirus (CoV) infection at molecular level. Treatment with quercetin inhibited SARS-CoV entry into the cells [123]. Molecular docking analysis studies indicated that quercetin can bind to SARS-CoV-2 proteins, such as spike (S) protein, chymotrypsin-like protease (3CL^pro^) main protease (M^pro^) protease, hich process the viral polypeptide to individual proteins and viral RNA-dependent RNA polymerase (RdRp) [116] with high affinity. Molecular docking studies also indicated quercetin may also inhibit activity of viral proteases and RNA polymerase [119]. Quercetin also binds to cellular proteins that bind to viral S protein and such as angiotensin converting enzyme 2 (ACE2) and transmembrane protease serine 2 (TMPRSS2) [117,118]. Such interactions can potentially inhibit viral proteases. Subsequently, quercetin was shown to inhibit SARS-CoV-2 replication and prevent syncytia formation in vitro [120]. In pilot clinical trials, quercetin treatment of SARS-CoV-2-infected patients with mild to moderate symptoms showed faster recovery, lower levels of serum lactate dehydrogenase and enhanced clearance of viral compared to placebo treated patients [121,122]. These findings indicate that quercetin may be beneficial when used as complementary agent for treating SARS-CoV-2 infection at early-stage.

In addition to blocking the entry of viruses, quercetin also reduces RSV-induced lung inflammation by unique mechanisms. In a mouse model of RSV infection, quercetin ameliorated lung inflammation, by down regulating IFN-γ, IL-1β, Il-6, TNF-α. Ex vivo, alveolar macrophages isolated from lungs of RSV-infected mice and treated with quercetin showed increase in itaconic acid through activation of immune responsive gene 1 and inhibited succinate dehydrogenase activity ultimately promoting polarization of macrophage from pro-inflammatory M1 to anti-inflammatory M2 [124]. Another study conducted metabolomics on RSV-infected mice and demonstrated that quercetin may limit RSV-induced inflammation through modulating purine metabolism pathways [125].

Quercetin also inhibits viral entry and replication of rhinoviruses, which is responsible for majority of common colds and is associated with acute exacerbations in patients with COPD and asthma. Quercetin inhibits PI-3 kinase-mediated endocytosis/entry of rhinovirus in airway epithelial cells, abrogates virus-induced cleavage of eIF4GI and increases phosphorylation of eIF2α [22]. Cleavage of eIF4GI inhibits initiation complex required for host cap-dependent protein synthesis and promotes viral protein synthesis. Phosphorylation of eIF-2α limits mRNA translation globally, which is essential to inhibit viral replication.

Oxidative stress and inflammatory responses are important factors that contribute to human metapneumovirus pathogenesis. Quercetin negatively modulates NF-κB and IRF-3 pathways to suppress metapneumovirus-induced oxidative stress and lung inflammation [113]. Recently, quercetin based nano formulation was demonstrated to effectively decrease viral titers and inflammatory markers in vivo and in vitro [126].

These observations suggest that quercetin exerts its protective role against respiratory viral infections by various mechanisms from preventing entry to limiting inflammation by modulating host innate immune responses.

## 4. Quercetin in COPD

### 4.1. Pathophysiology of COPD and Available Treatments

The exact mechanism of COPD pathogenesis is not completely understood but it is believed that the cigarette smoke and other environmental hazards induced persistent oxidative stress and lung inflammation contributes to the development of COPD [38,39,41]. Repeated airway epithelial injury and repair lead to abnormal airway epithelium which produces excessive levels of pro-inflammatory factors to recruit monocytes, neutrophils, lymphocytes and dendritic cells into the airways and lung parenchyma. Airway epithelial cells also release tissue degrading matrix metalloproteases (MMP). In addition, neutrophils and immature macrophages derived from recruited monocytes also release elastase and MMPs respectively. Both MMPs and elastases degrade elastin in the alveolar septa, and the elastin fragments further increase recruitment of inflammatory cells into the lungs.

Oxidative stress is markedly increased in COPD and this happens due to combination of exposure to exogenous oxidant radicals and endogenous reactive oxygen species released from activated neutrophils and macrophages. Further the oxidative stress is exacerbated by attenuated expression of antioxidant enzymes in the lungs [37]. The oxidative stress can further increase lung inflammation, mucus production, resistance to corticosteroids, and premature cell senescence.

Acute exacerbations in COPD are characterized by increase in respiratory symptoms which are sometimes accompanied by fever. Most of the acute exacerbations are due to respiratory infections which increases recruitment of inflammatory cells into the lungs, thus further increasing the lung inflammation and oxidative stress leading to progression of lung disease [127].

Current therapies for COPD are targeted for managing the symptoms but not the root cause of COPD that is eliminating oxidative stress and reducing inflammation. Therefore, quercetin with its potent antioxidant, anti-inflammatory and antimicrobial effects may be beneficial in COPD.

### 4.2. Effect of Quercetin in COPD

There are very few studies exploring the beneficial effects of quercetin in COPD despite its potent antioxidant and anti-inflammatory properties. We showed that in elastase/LPS-induced mouse model of COPD, oral treatment with quercetin reduces oxidative stress, suppresses pro-inflammatory cytokine production, and inhibits matrix metalloproteinases, particularly MMP-9 and MMP-12, which are key mediators of extracellular matrix degradation and emphysema development [128]. In cigarette smoke exposed COPD mouse model, mice on quercetin containing diet showed attenuated lung inflammation and this was associated with lower expression of CXCL-1, TNF-α, reduced infiltration of neutrophils and macrophages [129]. In another study, mice exposed to cigarette smoke for 5 days and treated with 10 mg/kg body weight quercetin by oral gavage reduced levels of leukocytes, oxidative stress, prevented changes in alveolar structure and lung function [130]. The same study also found that quercetin reduces cigarette smoke extract induced nitric oxide in mouse macrophage cells line. Recently, quercetin was also demonstrated to reduce infiltration of inflammatory cells, oxidative damage, cytokine levels and improve respiratory mechanics in mice exposed to cigarette smoke for 60 days. In another study, quercetin was demonstrated to mitigate airway remodeling by modulating autophagy through regulation of Wnt5a/β-catenin signaling pathway, reducing oxidative stress, alleviating mitochondrial damage and influencing phenotypic switch in alpha smooth muscle cells [131]. A recent study examined the effect of lipid-based quercetin formulations in a mouse model of COPD. This study used intratracheal administration rather than oral treatment and found that quercetin reduces cigarette smoke induced lung inflammation, oxidative stress, airway fibrosis, and improve lung function [132]. Quercetin also decreased the apoptotic ratio in mouse lungs by decreasing caspase 3 and caspase 7. These effects of quercetin were associated with suppression of NLRP3/IL-1β inflammasome pathway and the TGF-β1-related fibrosis signaling pathway. All these observations from preclinical models strongly indicate that quercetin may exert its therapeutic effects by reducing oxidative stress and inflammation, which are thought to play an important role in the pathogenesis of COPD.

Rhinovirus causes majority of common colds in normal subjects. But in patients with COPD, it causes acute exacerbation and this is often associated with severe respiratory symptoms [48,49,50,51]. In a preclinical model of COPD, infection with rhinovirus caused progression of lung disease, which is associated with further increases in infiltration of inflammatory cells, levels of pro-inflammatory cytokines, lung inflammation, airway remodeling, goblet cell metaplasia, and emphysema [133]. Mice consuming quercetin containing diet after rhinovirus infection showed significant reduction in rhinovirus-induced airway remodeling, goblet cell metaplasia, lung inflammation, accumulation of inflammatory macrophages and emphysema. Quercetin also improved lung mechanics, such as compliance and elastance in these mice [129]. Although this is the only study to show therapeutic effects of quercetin in acute exacerbation model, this study indicates that quercetin may prevent progression of lung disease following acute exacerbations associated with respiratory viral infections.

Airway epithelium in COPD patients is abnormal and show less ciliated cells, goblet cell metaplasia, and basal cell hyperplasia. Regenerated airway epithelium from COPD airway stem cells recapitulates all these features and shows pro-inflammatory phenotype [134]. Treatment of mucociliary-differentiated COPD cell cultures with quercetin decreased the levels of IL-8 by increasing the activity of forkhead transcription factor(FOX)O3A, which maintains the redox balance by increasing expression of antioxidant enzymes [11]. Interestingly, we also observed that brief treatment of COPD airway basal cells with 1 µM quercetin increased transepithelial electrical resistance (TEER), regeneration of airway epithelium with reduction in number of goblet cells, increase in ciliated cells and attenuated of IL-8 and IL-6 secretion [135]. This was associated with expression of several developmental genes including *homeobox* (*HOX*)*B2*, *E74-like factor* (*ELF*)*3*, *ELF5* and others. Further, treatment of COPD patients with quercetin for 6 months increased the expression of 2 developmental genes HOXB2 and ELF3. Downregulation of *HOXB2* and *ELF3* in COPD may occur because of epigenetic changes due to persistent exposure to inflammatory milieu. Since quercetin has been shown to modulate epigenetic marks by decreasing the activity of DNA methyltransferases, histone deacetylases and histone methyltransferases [27], it is plausible that quercetin may enhance expression of *HOXB2* and *ELF3* by modulating the epigenetic changes. Following rhinovirus infection COPD airway epithelial cell cultures show further increase in goblet cell metaplasia [136]. Brief treatment with 0.1 to 1 µM quercetin prevents RV-induced goblet cell metaplasia in these cultures by inhibition of NOTCH3 expression and activation. These results suggest that treatment with quercetin not only enhances repair/regeneration of airway epithelium from airway stem cells, but also improves innate immune function, potentially contributing to prevention of airway remodeling and progression of lung disease in COPD.

### 4.3. Clinical Trials of Quercetin in COPD

The increased intake of quercetin as dietary product from foods like apple and orange has been reported to reduce the incidence of asthma in Finland [137]. The flavonoids from apple have been reported to be beneficial for COPD patients by being positively associated with FEV_1_ and negatively associating with cough [138]. Apart from the natural forms, supplementation of pure quercetin in aglycone form along with vitamin C and niacin as oral administration in COPD patients for one week in a dose escalation manner of 500, 1000, or 2000 mg/day showed that quercetin is safely tolerated up to 2000 mg/day which was evaluated by blood profile and lung function questionnaire [139]. Recently we completed a small Phase II clinical trial examining the biological effects of quercetin in COPD patients [34]. In this study, COPD patients were treated with 2000 mg of quercetin/day or placebo for 6 months, blood and bronchoalveolar lavage fluid (BALF) were collected before and after treatment to detect markers of inflammation and oxidative stress. Due to the relatively small sample size, and very high variability in the examined markers between the patients at baseline, we compared baseline vs. after treatment and calculated the percent change from baseline. We found a significant reduction in the levels of 8-isoprostane (marker of oxidative stress), IL-8 and IL-1β, CXCL-10 (markers of inflammation) in BAL fluid in patients treated with quercetin, but not placebo-treated patients. The serum levels of surfactant protein D (SP-D) decreased significantly in quercetin-treated patients. SP-D is primarily produced in the lungs and the levels of SP-D in the blood correlate with lung damage. Quercetin, particularly in active smokers also increased the levels of anti-inflammatory molecule, CC16, and showed improvement in lung function. There was no change in the levels of C-reactive protein or 8-isoprostane in the serum. Interestingly, 7 out of 9 patients in quercetin group reported improvement in their respiratory symptoms. These data indicate that quercetin may reduce oxidative stress and inflammation in the lung compartment, but not systemic compartment. It is possible that longer than 6 months’ treatment may be required to see changes in systemic compartment. A larger clinical trial should be conducted to confirm the biological effects and clinical impact of quercetin in COPD.

## 5. Comparison with Current Therapy

Current pharmacological management of COPD primarily focuses on symptom control, reduction of exacerbations, and prevent progression of lung disease in COPD. Standard therapies used to control the respiratory symptoms are long-acting bronchodilators such as β2 agonists (LABA) or muscarinic antagonists (LAMA) for majority of the patients and both LABA/LAMA for patients with severe symptoms regardless of history of exacerbations [140,141]. Inhaled corticosteroids are also used in addition to LABA/LAMA, particularly in patients with eosinophilic inflammation or history of asthma. Roflumilast is a selective phosphodiesterase (PDE)4 inhibitor that targets systemic inflammation and is used in patients with severe COPD to reduce inflammation and prevent acute exacerbations [142]. However, its use is limited by significant adverse effects including intractable diarrhea, acute pancreatitis and weight loss [143]. Ensifentrine, a recently approved drug is dual PDE3/PDE4 inhibitor with a greater potency against PDE3 and has been developed as inhaled nebulized therapy. Because of its localized delivery, ensifentrine is associated with fewer systemic side effects than roflumilast and has clinical bronchodilator action with improvements in symptoms, and improve lung function, reduce exacerbations particularly in patients with <300 eosinophils/µL of blood [144,145]. Dupilumab is a monoclonal antibody targeting IL-4 receptor α subunit thereby blocking both IL-4 and IL-13 signaling. It is primarily used as a management therapy in COPD patients with type 2 inflammation as indicated by blood eosinophil counts >300/µL and are high risk for acute exacerbations. Dupilumab improves lung function, patient-reported respiratory symptom scores and reduces exacerbations [146]. However, all these therapies are expensive, have significant side effects and do not address the root cause of the diseases [143,144,146,147,148,149].

In contrast to these approved pharmacological therapies, quercetin exhibits a broader and more integrated mechanism of action by simultaneously targeting oxidative stress, inflammation, epithelial dysfunction, and bacterial and viral infections. Quercetin suppresses various inflammatory signaling pathways primarily by competing for ATP binding site in lipid and protein kinases, activity of which requires ATP. Quercetin reduces oxidative stress not only by scavenging free radicals, but also by promoting the expression of antioxidant enzymes via activation of Nrf2 and FOXO3A. In addition, quercetin may also improve airway epithelial structure and function by increasing the expression of proteins that are involved in airway epithelial repair. Daily treatment with quercetin may also prevent viral and bacterial infections given its antiviral and antibacterial properties. Moreover, quercetin is well-tolerated in COPD patients, less expensive with least side effects.

## 6. Conclusions and Future Directions

The use of quercetin may represent a promising therapeutic approach for mitigating COPD and its associated complications. Its potential benefits arise from a multifaceted mode of action, including the prevention of viral infections, attenuation of COPD-associated inflammation, and reduction of oxidative stress. By targeting these interconnected pathological processes, quercetin may not only alleviate the clinical symptoms of COPD but also be useful in preventing disease progression.

However, despite these promising properties, important limitations remain. The clinical translation of quercetin is hindered by chemical instability, poor bioavailability, rapid metabolism, and variability in absorption. Given that quercetin also improves heart health, reduces glucose levels, inhibits PDE, has anticancer effects in addition to its antioxidant, anti-inflammatory and antiviral activities, it may become an essential treatment for COPD patients who have multiple co-morbidities. Future studies should be directed towards developing formulations, that can maintain the stability of the quercetin, enhance water solubility of quercetin, improve bioavailability and safely-tolerated in COPD patients. The clinical trials with quercetin in patients with chronic diseases is often done with concomitant medications. To establish the beneficial effects of quercetin, a larger clinical trial with improved quercetin formulation with and without concomitant medications is needed. Quercetin may potentially be more effective in patients with mild COPD and may prevent progression of lung disease by inhibiting oxidative stress, and inflammatory pathways which are the main culprits in COPD development/disease progression. Therefore, a clinical trial with quercetin in patients with mild COPD is warranted. The future studies should also focus on identification of suitable and more robust blood and/or nasal biomarkers to monitor the effects of quercetin in addition to lung function and respiratory symptoms.

## Figures and Tables

**Table 1:** Pharmacokinetics of quercetin in its natural source, quercetin aglycone, derivatives of quercetin and quercetin formulations.

Study Design Product/Source and (Ref)	Dose (mg/d)	Number of Subjects	T_max_ (h)	T_1/2_ (h)	C_max_ (ng/mL)	AUC_(0-Variable in h)_	AUC (µg·h/mL)
Multiple dose [85]							
Quercetin aglycone	500	*n* = 10	3	3.47	15.4	(0–8)	62.5
Multiple dose [89]		*n* = 15					
Quercetin aglycone	50	*n* = 5	2	16.5	189	(0–8)	49.3
	100	*n* = 5	3	14.1	295	(0–8)	65.7
	150	*n* = 5	6	19.8	431	(0–8)	99.2
Single dose Random Crossover [88]		*n* = 9					
Onions	68		0.7 ± 1.1	28 ± 92	224 ± 44	(0–36)	2.33 ± 8.5
Apples	98		2.5 ± 0.7	23 ± 32	92 ± 19	(0–36)	1.06 ± 3.7
Quer-3-rutinoside	100		9.3 ± 1.8	Not presented	90 ± 93	(0–36)	0.98 ± 9.9
Single dose, random crossover [72]		*n* = 9					
Quer-4-glucoside	154		0.61 ± 0.2	17.7 ± 0.9	1345 ± 212	(0–72)	5.28 ± 7.3
Quer-3-glucoside	151		0.45 ± 0.08	18.5 ± 0.8	1526 ± 315		5.78 ± 8.7
Single dose, random crossover [73]		*n* = 12					
Onion Supplement	200		0.68 ± 0.22	10.9 ± 4.1	2310 ± 1460	(0–24)	9.7 ± 6.9
Buckwheat tea Powder	200		4.32 ± 1.83	10.3 ± 3.5	640 ± 670	(0–24)	3.8 ± 3.9
Quer-4′-O-glucoside	100		0.70 ± 0.31	11.9 ± 4.0	2120 ± 1630	(0–24)	8.4 ± 9.1
Quer-3-rutinoside	200		6.98 ± 2.94	11.8 ± 3.1	320 ± 340	(0–24)	2.5 ± 2.2
Single dose [86]		*n* = 18					
Quercetin + tang	500	*n* = 6	4.7 ± 0.3	8.3 ± 1.4	354.4 ± 87.6	(total)	3.84 ± 0.69
First strike^TM^ Bars	500	*n* = 6	2.3 ± 0.5	8.0 ± 1.3	698.1 ± 189.5	(total)	5.38 ± 1.43
RealFX-Q plus chews	500	*n* = 6	3.3 ± 0.8	5.5 ± 0.9	1051.9 ± 393.1	(total)	4.15 ± 0.67
Single dose random crossover [81]		*n* = 9					
Quercetin	500		4.83 ± 0.52	6.26 ± 1.25	10.93 ± 2.22	(0–24)	4.77 ± 1.19
Quercetin phytosome	250		3.81 ± 0.60	3.36 ± 0.22	126.3 ± 14.8	(0–24)	50.40 ± 6.42
Quercetin phytosome	500		3.38 ± 0.61	3.78 ± 0.14	223.10 ± 16.32	(0–24)	96.16 ± 9.29

Shaded rows represent individual Studies indicating study design that is single dose vs. multiple dose, random crossover vs. individual cohort, and a total number of patients in the study.

**Table 2:** Effect of Quercetin on viral infection.

Quercetin Dose (Ref)	Experimental Model	Route of Administration	Target Virus	Effect of Quercetin/Key Molecular Target
0.2 mg of quercetin [22]	Mice	Intranasal route and gavage	Rhinovirus	Post treatment administration of quercetin reduced viral load, IL-8 and IFN responses in airway epithelial cells.
6.25 mg/kg quercetin 3-rhamnoside (Q3R) [109]	mice	Oral	Influenza A virus	Q3R administration on influenza-infected mice showed moderate inflammation including pulmonary edema and elevated numbers of inflammatory cells and necrosis and hampered the progression and development of pulmonary lesions and postinfection pulmonary edema.
Quercetin [111]	mice	Oral	influenza virus A H3N2 virus	Quercetin significantly increases the pulmonary concentrations of catalase, reduced glutathione, and superoxide dismutase.
10 mg/L quercetin [112]	A549 cells	N/A	H1N1 influenza virus	Quercetin treatment inhibited the H1N1 cytopathic effect on A549 cells by reducing the CDK4 levels.
5.17 µg/mL Quercetin-7-O-glucoside [108]	MDCK cells	N/A	Influenza A and B virus	Q7G showed strong inhibition activity against influenza A and B viruses by inhibiting viral RNA polymerase. It occupies the binding site of m(7)GTP on viral PB2 protein and reduces virus-induced ROS and autophagy.
Quercetin [113]	A549 cells	N/A	Human metapneumovirus	Quercetin significantly reduced cellular oxidative damage, viral replication, and inflammatory mediator secretion. It inhibited the transcription factor nuclear factor (NF)-κB and interferon regulatory factor (IRF)-3 binding to promoter region of their target genes.
Quercetin pentaacetate [114]	Hep2 cells	N/A	Respiratory Syncytial virus	Quercetin interacts with F-protein on hRSV surface to inhibit its adhesion. This virucidal effect of quercetin occurs during adhesion step of cycle hRSV replication.
Acetylated derivatives of Quercetin [115]	In vitro	N/A	Respiratory Syncytial viral protein	It interacts with the RNA-binding sites of M2-1 responsible for viral replication.
Quercetin, Quercetin-3-O-glucoside [116,117,118,119]	Molecular docking	N/A	SARS-CoV-2	Binds to viral S, 3CL^pro^, M^pro^ and RdRp and host proteins ACE2 and TMPRSS2 with high affinity. Inhibits viral proteases and polymerase and ACE2.
Quercetin [120]	Vero2 and Caco2 cells	N/A	SARS-CoV-2	Quercetin inhibits replication of virus and prevents syncytium formation.
Quercetin [121,122]	Clinical trial	Human	SARS-CoV-2	SARS-CoV-2 infected patients with mild to moderate symptoms treated with quercetin showed faster recovery than placebo treated patients.

N/A: not applicatble.

## Data Availability

No new data were created or analyzed in this study. Data sharing is not applicable to this article.

## Abbreviations

The following abbreviations are used in this manuscript:

AP-1	Activator Protein-1
ARE	Antioxidant Response Element
ASMCs	Airway Smooth Muscle Cells
ATP	Adenosine Triphosphate
CCL	C-C Motif Chemokine Ligand
CC16	Club Cell Secretory Protein 16
CDK4	Cyclin-Dependent Kinase 4
COPD	Chronic Obstructive Pulmonary Disease
COX-2	Cyclooxygenase-2
CRP	C-Reactive Protein
CXCL	C-X-C Motif Chemokine Ligand
DPPH	2,2-Diphenyl-1-picrylhydrazyl
DNMTs	DNA Methyltransferases
ECM	Extracellular Matrix
EGFR	Epidermal Growth Factor Receptor
ELF3	E74 Like ETS Transcription Factor 3
ETC	Electron Transport Chain
FEV1	Forced Expiratory Volume in 1
GCL	Glutamate-Cysteine Ligase
GM-CSF	Granulocyte-Macrophage Colony-Stimulating Factor
GSH	Glutathione
GST	Glutathione S-Transferase
H1N1	Hemagglutinin Type 1 Neuraminidase Type 1
H3N2	Hemagglutinin Type 3 Neuraminidase Type 2
HDACs	Histone Deacetylases
HO-1	Heme Oxygenase-1
HOXB2	Homeobox B2
HMTs	Histone Methyltransferases
ICAM-1	Intercellular Adhesion Molecule-1
IFN-γ	Interferon Gamma
IL	Interleukin
iNOS	Inducible Nitric Oxide Synthase
IRAK1	Interleukin-1 Receptor-Associated Kinase 1
IRF-3	Interferon Regulatory Factor 3
LDH	Lactate Dehydrogenase
LPS	Lipopolysaccharide
MMP	Matrix Metalloproteinase
MMP-9	Matrix Metalloproteinase-9
MMP-12	Matrix Metalloproteinase-12
MDCK	Madin-Darby Canine Kidney
NF-κB	Nuclear Factor Kappa B
NLRP3	NOD-, LRR-, and Pyrin Domain-Containing Protein 3
NQO1	NAD(P)H Quinone Dehydrogenase 1
NRF2/Nrf2	Nuclear Factor Erythroid 2–Related Factor 2
PB2	Polymerase Basic Protein 2
Q3R	Quercetin 3-Rhamnoside
Q7G	Quercetin-7-O-Glucoside
QQ	O-Quinone/Quinone Methide
RNA	Ribonucleic Acid
ROS	Reactive Oxygen Species
RSV	Respiratory Syncytial Virus
SARS-CoV-2	Severe Acute Respiratory Syndrome Coronavirus 2
SGLT1	Sodium-Glucose Cotransporter 1
SOD	Superoxide Dismutase
Src	Proto-Oncogene Tyrosine-Protein Kinase Src
Syk	Spleen Tyrosine Kinase
T2	Type 2
TGF-β	Transforming Growth Factor Beta
Th2	T Helper 2 Cells
TLR4	Toll-Like Receptor 4
TNF-α	Tumor Necrosis Factor Alpha
TSLP	Thymic Stromal Lymphopoietin
Wnt5a	Wingless-Related Integration Site 5A
